# Adrenocortical suppression and recovery after continuous hypnotic infusion: etomidate versus its soft analogue cyclopropyl-methoxycarbonyl metomidate

**DOI:** 10.1186/cc12494

**Published:** 2013-01-30

**Authors:** Rile Ge, Ervin Pejo, Joseph F Cotten, Douglas E Raines

**Affiliations:** 1 Department of Anesthesia, Critical Care, and Pain Medicine, Massachusetts General Hospital, 55 Fruit Street, Boston, MA 02114, USA

## Abstract

**Introduction:**

Etomidate is no longer administered as a continuous infusion for anesthetic maintenance or sedation, because it results in profound and persistent suppression of adrenocortical steroid synthesis with potentially lethal consequences in critically ill patients. We hypothesized that rapidly metabolized soft analogues of etomidate could be developed that do not produce persistent adrenocortical dysfunction even after prolonged continuous infusion. We hope that such agents might also provide more rapid and predictable anesthetic emergence. We have developed the soft etomidate analogue cyclopropyl-methoxycarbonyl etomidate (CPMM). Upon termination of 120-minute continuous infusions, hypnotic and encephalographic recoveries occur in four minutes. The aims of this study were to assess adrenocortical function during and following 120-minute continuous infusion of CPMM and to compare the results with those obtained using etomidate.

**Methods:**

Dexamethasone-suppressed rats were randomized into an etomidate group, CPMM group, or control group. Rats in the etomidate and CPMM groups received 120-minute continuous infusions of etomidate and CPMM, respectively. Rats in the control group received neither hypnotic. In the first study, adrenocortical function during hypnotic infusion was assessed by administering adrenocorticotropic hormone (ACTH) 90 minutes after the start of the hypnotic infusion and measuring plasma corticosterone concentrations at the end of the infusion 30 minutes later. In the second study, adrenocortical recovery following hypnotic infusion was assessed by administering ACTH every 30 minutes after infusion termination and measuring plasma corticosterone concentrations 30 minutes after each ACTH dose.

**Results:**

During hypnotic infusion, ACTH-stimulated serum corticosterone concentrations were significantly lower in the CPMM and etomidate groups than in the control group (100 ± 64 ng/ml and 33 ± 32 ng/ml versus 615 ± 265 ng/ml, respectively). After hypnotic infusion, ACTH-stimulated serum corticosterone concentrations recovered to control values within 30 minutes in the CPMM group but remained suppressed relative to those in the control group for more than 3 hours in the etomidate group.

**Conclusions:**

Both CPMM and etomidate suppress adrenocortical function during continuous infusion. However, recovery occurs significantly more rapidly following infusion of CPMM.

## Introduction

**Figure 1:**
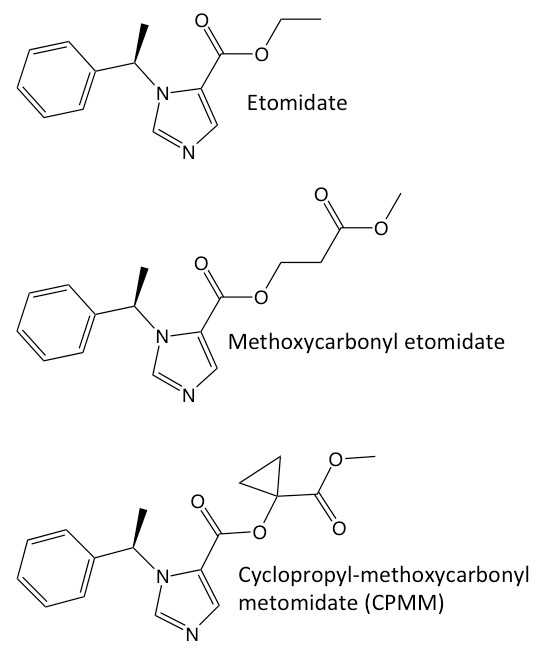
**Chemical structures of etomidate, methoxycarbonyl etomidate and cyclopropyl-methoxycarbonyl etomidate**.

Etomidate is an imidazole-based intravenous sedative-hypnotic (Figure 1). It was developed by Janssen Pharmaceuticals in the 1960s and introduced into clinical practice as an anesthetic induction and maintenance agent a decade later [1]. Since its introduction, widespread concerns regarding its use, particularly in the critically ill, have arisen because it produces profound and persistent adrenocortical suppression by inhibiting 11β-hydroxylase [2-10].

Considering the hypothesis that rapidly metabolized soft analogues of etomidate that would not produce persistent adrenocortical suppression could be designed, our laboratory developed methoxycarbonyl etomidate [11]. Similar to remifentanil and esmolol, methoxycarbonyl etomidate is rapidly metabolized by esterases to a carboxylic acid metabolite. As a hypnotic and inhibitor of adrenocortical steroid synthesis, this metabolite is two to three orders of magnitude less potent than the parent compound [12]. When given as an intravenous bolus to rats, methoxycarbonyl etomidate is an extremely short-acting hypnotic agent that maintains hemodynamic stability and does not produce prolonged adrenocortical suppression [11].

Subsequent continuous infusion studies demonstrated that the very rapid metabolism of methoxycarbonyl etomidate, along with its relatively low potency, necessitated very high dosing to maintain hypnosis [13]. This led to the accumulation of metabolite, and concentrations in the blood and cerebrospinal fluid reached the millimolar levels sufficient to produce pharmacological effects that persisted for hours. We have now developed a second-generation soft etomidate analogue, cyclopropyl-methoxycarbonyl etomidate (CPMM), which is more potent and longer-lasting than methoxycarbonyl etomidate but still more rapidly metabolized than etomidate [14]. Unlike with methoxycarbonyl etomidate or etomidate, hypnotic and encephalographic recoveries occur in 4 minutes independently of infusion duration [15]. The aims of this study were to assess adrenocortical function during and after 120-minute continuous infusion of CPMM and to compare the results with those obtained by using etomidate.

## Materials and methods

### Animals

All studies were conducted with the approval of the Institutional Animal Care and Use Committee at Massachusetts General Hospital (Boston, MA, USA) and in accordance with its rules and regulations. Adult male Sprague-Dawley rats (250 to 500 g) were purchased from Charles River Laboratories (Wilmington, MA, USA) and housed in the Massachusetts General Hospital Center for Comparative Medicine animal care facility. All intravenous drugs were administered through a femoral venous catheter pre-implanted by the vendor prior to animal delivery to our animal care facility. Each rat was individually caged with controlled temperature (21 ± 2°C) and light-dark cycles (7 a.m. to 7 p.m.) in the Massachusetts General Hospital Center for Comparative Medicine animal care facility, and food and water were provided *at libitum*.

### Drugs

Etomidate (2 mg/mL in 35% propylene glycol/water) was from Hospira (Lake Forest, IL, USA). CPMM was synthesized (> 97% purity) by Aberjona Laboratories (Beverly, MA, USA) by using our previously described approach [14] and solubilized at 5 mg/mL in 10% propylene glycol/normal saline. Dexamethasone was purchased from American Regent (Shirley, NY, USA), and adrenocorticotropic hormone (ACTH) was from Sigma-Aldrich (St. Louis, MO, USA).

### Hypnotic drug infusion protocol

Our goals were to achieve and maintain approximately equivalent anesthetic depths during 120-minute hypnotic infusions. Therefore, for each hypnotic, we used a continuous infusion protocol that, as we previously determined [15], achieves the following behavioral endpoints: (a) it maintains the electroencephalographic burst suppression ratio at 80% (in a background of 1% isoflurane) and (b) it administers a steady-state dose that is 1.3 times that required to produce immobility to a standard noxious stimulus (in the absence of isoflurane). The total doses of CPMM and etomidate delivered during 2-hour infusions were 143 and 36 mg/kg, respectively. During hypnotic infusion, the body temperature of each rat was measured with a rectal thermistor and maintained at 37 ± 0.2°C by using a heat lamp. Rats in the control group received dexamethasone but no hypnotic during the hypnotic infusion protocol and were allowed to roam freely in their cages.

### Assessment of adrenocortical function during hypnotic drug infusion

**Figure 2:**
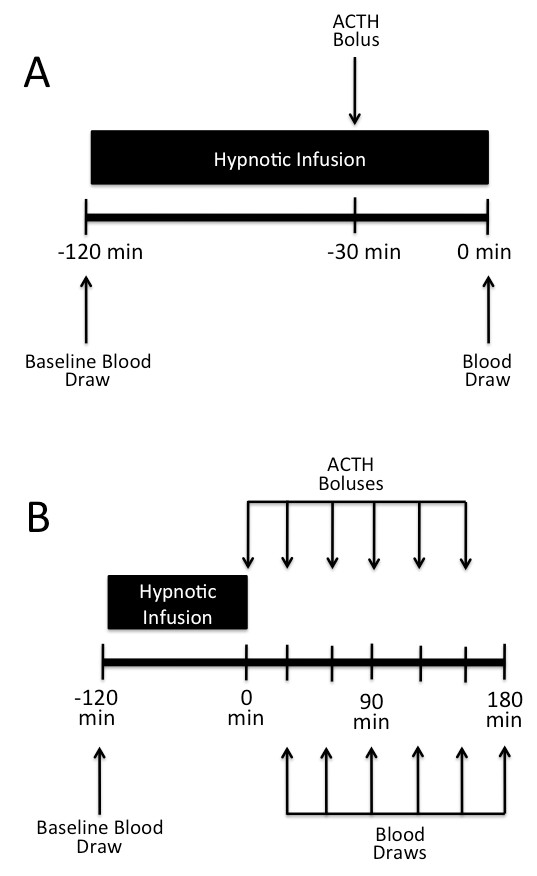
**Schematic diagrams depicting the protocols used for assessing adrenocortical function (a) during or (b) after hypnotic infusion**. (a) A baseline blood sample was drawn and the hypnotic infusion was begun. Ninety minutes after beginning the infusion, adrenocorticotropic hormone (ACTH) was administered and a second blood sample was drawn at the end of the infusion 30 minutes later. (b) A baseline blood sample was drawn and the hypnotic infusion was begun. Upon completion of the 120-minute hypnotic infusion, ACTH dose was repetitively administered and a blood sample was drawn every 30 minutes. For clarity, dexamethasone administration is not shown.

Rats were given intravenous dexamethasone (0.2 mg) to suppress corticosterone synthesis elicited by endogenous ACTH and reduce baseline corticosterone concentrations. Rats were then randomly assigned to CPMM, etomidate, or control groups. Two hours after dexamethasone administration, a baseline blood sample was drawn, a second dose of dexamethasone was given, and the hypnotic infusion protocol was begun. Ninety minutes after the protocol began, ACTH (0.25 mg/kg) was given intravenously to stimulate corticosterone production, and a second blood sample was drawn 30 minutes later (that is, at the end of the infusion protocol). Thus, the corticosterone concentration in the second blood sample was a measure of the extent of adrenocortical inhibition during the final 30 minutes of the infusion (Figure 2A).

### Assessment of adrenocortical recovery after hypnotic drug infusion

Rats were given intravenous dexamethasone (0.2 mg) and randomly assigned to CPMM, etomidate, or control groups. Two hours after dexamethasone administration, a baseline blood sample was drawn, a second dose of dexamethasone was given, and the hypnotic infusion protocol was begun. After the 120-minute infusion protocol was completed, ACTH (0.25 mg/kg) was given intravenously every 30 minutes for 3 hours to maintain adrenocortical stimulation, and blood samples were drawn 30 minutes after each ACTH dose. The time course of adrenocortical recovery was assessed from the time-dependent corticosterone concentration increase that occurred with repetitive ACTH administration (Figure 2B).

### Measurement of serum corticosterone concentrations

Serum corticosterone concentrations in blood samples were determined as previously described [11] by using an enzyme-linked immunosorbent assay (Diagnostic Systems Laboratories, Webster, TX, USA) and a 96-well plate reader (Molecular Devices, Sunnyvale, CA, USA).

### Statistical analysis

All data are presented as mean ± standard deviation. Statistical analyses were carried out by using Prism version 5.0 for the Macintosh (GraphPad Software, Inc., La Jolla, CA, USA). For multiple comparisons, we performed either a one-way or two-way analysis of variance followed by a Bonferroni post-test. A *P *value less than 0.05 was considered statistically significant.

## Results

### Suppression of adrenocortical function during hypnotic drug infusion

**Figure 3:**
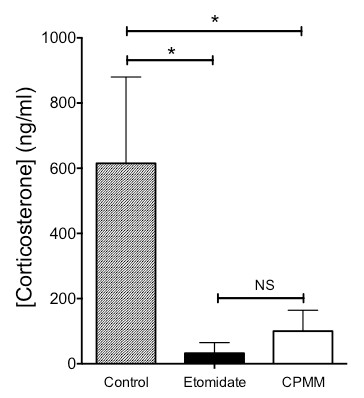
**Adrenocorticotropic hormone-stimulated serum corticosterone concentrations during 120-minute infusions**. Serum corticosterone concentrations in the cyclopropyl-methoxycarbonyl etomidate (CPMM) and etomidate groups were 100 ± 64 ng/mL and 33 ± 32 ng/mL, respectively, versus 615 ± 265 ng/mL in the control group. *P < 0.001. Rats in the control group received neither CPMM nor etomidate during the hypnotic infusion period. Six rats were used in each group. NS, not significant.

Baseline serum corticosterone concentrations prior to the start of the infusion protocol were not significantly different among the three groups and averaged 37 ± 111 ng/mL. After administration of ACTH, serum corticosterone concentrations in the CPMM and etomidate groups were 100 ± 64 ng/mL (*n *= 6) and 33 ± 32 ng/mL (*n *= 6), respectively (Figure 3). These values were not significantly different from one another but were significantly lower than that measured in the control group (615 ± 265 ng/mL, *n *= 6, *P *< 0.001).

### Recovery of adrenocortical function after hypnotic drug infusion

**Figure 4:**
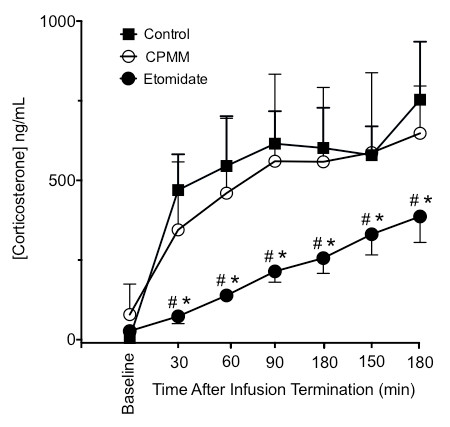
**Adrenocorticotropic hormone (ACTH)-stimulated serum corticosterone concentrations following 120-minute infusions**. The baseline blood sample was drawn immediately prior to beginning the hypnotic infusion and before administering ACTH. ACTH was first administered at time 0 and then repetitively administered every 30 minutes. Serum corticosterone concentrations were measured in blood drawn 30 minutes after each ACTH dose. The indicated times are when blood was drawn *P < 0.05 versus control group. ^#^*P *< 0.05 versus cyclopropyl-methoxycarbonyl etomidate (CPMM) group. Six rats were used in each group.

Baseline serum corticosterone concentrations prior to the start of the infusion protocol or administration of ACTH were not significantly different among the three groups and averaged 63 ± 108 ng/mL. With ACTH administration, serum corticosterone concentrations increased over time in all three groups (Figure 4). At every time point of the study, ACTH-stimulated serum corticosterone concentrations in the CPMM and control groups were not significantly different. In contrast, ACTH-stimulated serum corticosterone concentrations in the etomidate group were significantly lower than in either the CPMM or control groups for all time points.

## Discussion

This study shows that, during 120-minute continuous infusions of CPMM and etomidate, ACTH-stimulated serum corticosterone concentrations were significantly reduced relative to the control value. However, after the infusion was completed, ACTH-stimulated serum corticosterone concentrations were not significantly different from control values within 30 minutes in rats that received CPMM, but remained significantly lower than control values for more than 3 hours in rats that received etomidate. We conclude that continuous infusions of CPMM and etomidate cause adrenocortical suppression but that adrenocortical recovery occurs more quickly after infusions of CPMM as compared with etomidate.

Suppression of adrenocortical function is a common side effect of etomidate administration that results from the inhibition of 11β-hydroxylase, an enzyme required for the biosynthesis of cortisol, corticosterone, and aldosterone [4,16-18]. This potentially deadly side effect has led anesthesiologists and intensivists to abandon its use by continuous infusion for anesthetic maintenance or sedation [3,19]. Although etomidate continues to be used as an anesthetic induction agent, significant concerns about the impact of even a single bolus dose remain because even this brief exposure produces long-lasting adrenocortical suppression that may be detrimental to the critically ill [6-10,20-22].

Why is adrenocortical function suppressed for so long after etomidate administration? The adrenocortical inhibitory potency of etomidate is two to three orders of magnitude greater than its hypnotic potency [23-27]. Thus, a typical anesthetic induction dose of etomidate represents a massive overdose with respect to adrenocortical inhibition, and etomidate concentrations in blood fall below those that produce adrenocortical suppression only after the passage of multiple elimination half-lives.

Our goal in creating soft etomidate analogues was to produce agents that may be given as either a bolus (for anesthetic induction) or continuous infusion (for anesthetic maintenance) without producing persistent adrenocortical suppression since they are rapidly eliminated. We hoped that such agents might also allow fast emergence from anesthesia, which might ultimately benefit patients while improving operational efficiencies. Our first soft etomidate analogue, methoxycarbonyl etomidate, achieved these goals when given as a single bolus since hypnotic recovery was rapid and adrenocortical suppression was brief [11]. However, because it was so rapidly metabolized and possessed relatively low hypnotic potency, dosing requirements during continuous infusion were extremely high [13]. As a consequence, metabolite accumulated during infusions and recovery times were highly dependent upon the duration of hypnotic infusion (that is, recovery was context-dependent).

With the goals of slowing methoxycarbonyl etomidate's exceptionally fast rate of metabolism and reducing the extent of metabolite accumulation during infusions, we modified the drug's chemical structure by adding a cyclopropyl group directly adjacent to the metabolically labile ester moiety (to sterically hinder enzymatic attack) and shortening the spacer between that ester and the etomidate core (Figure 1). This structural change increased both the duration of hypnotic action and hypnotic potency by approximately eightfold [14]. As a result, dosing requirements for continuous CPMM infusions are one to two orders of magnitude lower than those for methoxycarbonyl etomidate infusions, and neither hypnotic nor encephalographic recoveries are delayed by the accumulation of metabolite [14,15].

We believe that the introduction of CPMM into clinical practice could have a significant impact on patient care by allowing anesthesia to be maintained, even in older or critically ill patients, without producing significant cardiovascular depression or persistent adrenocortical dysfunction. It may also allow more rapid and predictable anesthetic emergence than is possible with currently available agents.

There are several limitations of our study. First is the use of a rat model to study adrenocortical recovery after hypnotic infusion. Rats often metabolize ester-containing drugs (including etomidate) faster than humans [28,29]. Therefore, adrenocortical recovery following infusion of CPMM and etomidate may be somewhat slower in humans than we observed in rats. Second, rats use corticosterone, rather than cortisol, as the primary stress steroid. Thus, we are studying a different steroid. However, because the biosynthesis of both steroids requires 11β-hydroxylase, suppression of corticosterone synthesis in rats is expected to be a relevant measure of adrenocortical function in the setting of etomidate or CPMM administration. Finally, we used a protocol for assessing the time course of adrenocortical recovery in which ACTH is administered multiple times, introducing the potential for adrenal fatigue toward the end of the study. Such fatigue would be greater for rats in the control and CPMM groups that are most actively producing steroids but would not alter our conclusions.

The development of soft etomidate analogues follows a trend in drug development toward agents that are ultra-short-acting and is in response to the need in the operating room and intensive care unit for more precise temporal control of drug effects [30]. Esmolol and remifentanil are commonly used examples of soft drugs that serve this need and use the same labile ester strategy employed in our etomidate analogues [31]. The esterase-metabolized soft calcium channel blocker clevidipine was recently introduced into clinical practice for the acute treatment of hypertension [32], and the esterase-metabolized soft midazolam analogue remimazolam is currently undergoing clinical trials [33]. By offering rapid emergence from anesthesia and prompt return of adrenocortical function, CPMM may join the growing list of soft drugs available for clinical use.

## Conclusions

Adrenocortical function was suppressed during 120-minute continuous infusions of CPMM and etomidate in a rat model. However, after infusion termination, adrenocortical recovery occurred in less than 30 minutes with CPMM but persisted for more than 3 hours with etomidate.

## Key messages

• Cyclopropyl-methoxycarbonyl etomidate (CPMM) is a rapidly metabolized analogue of etomidate.

• CPMM and etomidate suppress adrenocortical steroid synthesis during 120-minute continuous infusions.

• Upon termination of 120-minute continuous infusions, adrenocortical recovery occurs faster with CPMM (< 30 minutes) than with etomidate (> 3 hours).

• CPMM may be a viable alternative to other sedative hypnotics (for example, propofol, etomidate, and inhaled agents) in which intraoperative hemodynamic stability and rapid anesthetic recovery are desirable, and adrenocortical suppression persisting for little more than the duration of hypnotic administration is acceptable.

## Abbreviations

ACTH: adrenocorticotropic hormone; CPMM: cyclopropyl-methoxycarbonyl etomidate.

## Competing interests

Massachusetts General Hospital has submitted patent applications for soft etomidate analogues. Two authors (JFC and DER) and their respective laboratories, departments, and institutions could receive compensation related to the development or sale of the technology presented in this report. DER is a consultant for and holds an equity interest in Annovation BioPharma Inc. (Cambridge, MA, USA), which has licensed this technology for development. RG and EP declare that they have no competing interests.

## Authors' contributions

RG participated in the design of the study, carried out the data acquisition and analysis, and participated in drafting the manuscript. EP assisted with study design, data acquisition, and analysis and participated in drafting the manuscript. JFC developed and implemented the computer software to administer hypnotics and assisted with study design and drafting the manuscript. DER conceived and designed the study, carried out the coordination, performed the statistical analyses, and drafted the manuscript. All authors read and approved the final manuscript.
